# Rhein induces liver cancer cells apoptosis via activating ROS-dependent JNK/Jun/caspase-3 signaling pathway

**DOI:** 10.7150/jca.30381

**Published:** 2020-01-01

**Authors:** Aili Wang, Huihong Jiang, Yuanyuan Liu, Jing Chen, Xue Zhou, Chenxi Zhao, Xia Chen, Mobin Lin

**Affiliations:** 1 Center for clinical research and translational medicine, Yangpu hospital, Tongji University School of Medicine, Shanghai 200090, China.; 2 Institute of Gastrointestinal Surgery and Translational Medicine, Tongji University School of Medicine, Shanghai 200090, China.; 3 Department of General Surgery, Yangpu Hospital, Tongji University School of Medicine, Shanghai 200090, China.

**Keywords:** Rhein, liver cancer, apoptosis, ROS, JNK/Jun/caspase-3 signaling pathway

## Abstract

**Background:** Liver cancer is one of the leading cancers in China. Rhein induces apoptosis in various human cancer cells, but the underlying mechanism is still unknown.

**Methods:** In the present study, the MTT assay was used to detect the anti-cell growth ability of Rhein on liver cancer cells. Hoechst33342 staining and FACS assay were used to detect cell apoptosis. Finally, the effect of Rhein on JNK protein' phosphorylation level and the apoptosis-associated proteins were determined by western blot.

**Results:** Here, we found that Rhein significantly inhibited the cell viability in a dose-dependent and time-dependent manner both in HepG2 and Huh7 cells. Also, Rhein increased the apoptosis, mitochondrial membrane potential (MMP) and cell-cycle arrest. Furthermore, we observed that the ROS level and JNK/Jun/caspase-3 signaling pathway played a key role in Rhein induced apoptosis. Our study further demonstrated that Rhein increases apoptosis by inducing the generation of ROS and activating the JNK/Jun/caspase-3 signaling pathway.

**Conclusions:** The present study showed that Rhein promotes apoptosis via regulating ROS/JNK/Jun/caspase-3 signaling pathway both in HepG2 and Huh7 cells. Rhein may be a promising therapeutic candidate for the treatment of liver cancer.

## Introduction

Liver cancer, the third cancer-related cause of death, is one of the leading cancers with a rapid upward trend all over the world. Epidemiology studies have shown that liver cancer is the second most common digestive cancer in Asia. In China, there is a large variation in the incidence, with high incidence rates in men of more than 30 per 100,000 1. However, no chemotherapeutics have been evidenced better efficacious therapies and prevention strategies for liver cancer 1. Therefore, the identification of novel bioactive compounds with promising anticancer activity and investigation of their cellular targets remains needed.

**Figure 1:**
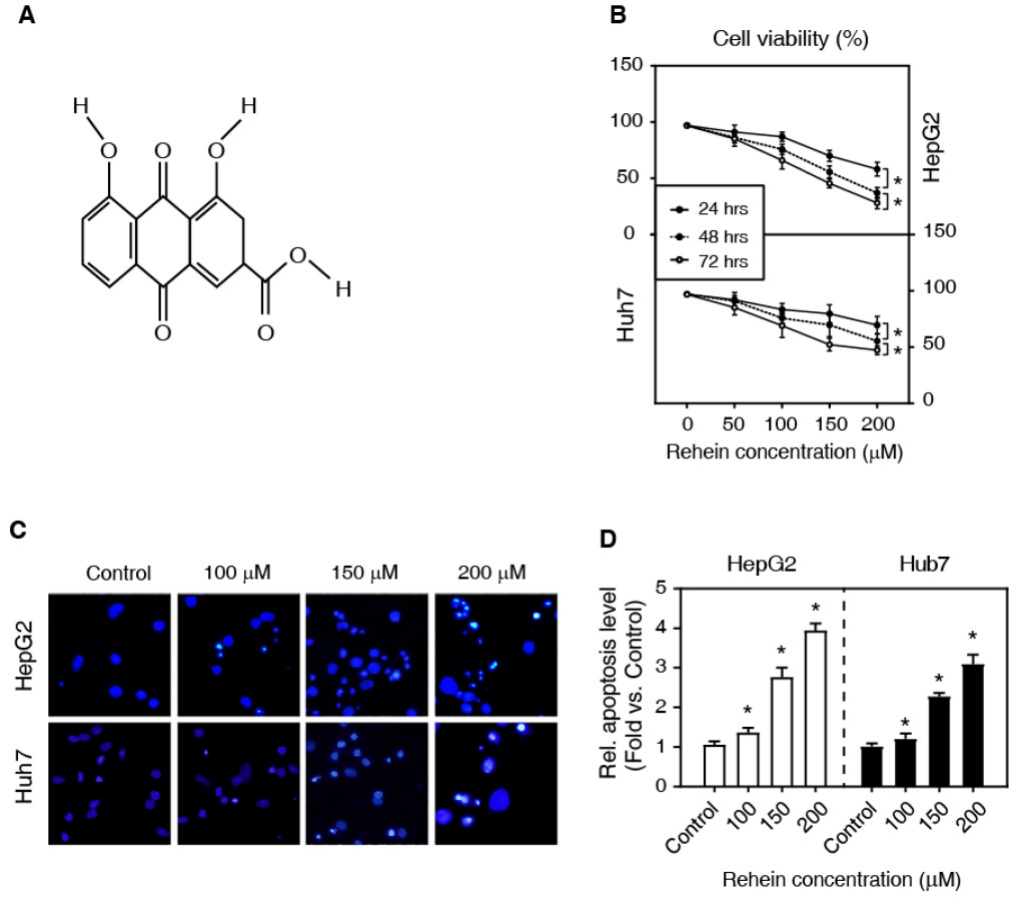
Rhein increased the apoptosis of HepG2 and Huh7 cells. **(A)** The chemical structure of Rhein. **(B)** HepG2 and Huh7 cells were treated with Rhein (0, 50, 100, 150 and 200 μmol/L) for 24, 48 and 72 h, respectively. Cell viability was measured by MTT assay. *P < 0.05. **(C, D)** HepG2 and Huh7 cells were treated with Rhein (0, 100, 150 and 200 μmol/L) for 24 h, and then the apoptotic morphological characteristics were stained with Hoechst 33342 staining. *P < 0.05 compared with the control group.

Rhein (4,5-dihydroxyanthraquinone-2-carboxylic acid, Figure 1A), a main constituent of rhubarb, is found in medicinal herbs including rheum palmatum l, cassia tora l, polygonum multiflorum thunb, aloe barbadensis miller, and so on. Rhein has been used medicinally for more than 1,000 years in China for the treatment of inflammatory diseases including osteoarthritis, diabetic nephropathy. The recent studies have demonstrated that Rhein induces significant apoptosis in human breast, colon, lung, and glioma cancer cell lines *in vitro* 2. Apoptosis, a physiological process for eliminating malignant cells including cancer cells, plays a key role in anticancer without damaging normal cells and tissues 3, 4. Previously, the substantial evidence showed that Rhein induces the cell cycle S-phase arrest and results in DNA fragmentation via complex mechanisms including decreasing Bcl-2 and cleaved caspase-3 level and increasing ROS and phosphorylated c-JNK level 2, 5, 6. These results suggest that Rhein induces apoptosis in various human cancer cells. However, whether Rhein is effective in killing liver cancer cells is still unknown.

Based on the above considerations, the aim of our present study is to investigate the potential anticancer effects of Rhein on hepatoma cells including HepG2 and Huh7 cells, and to further explore the underlying molecular mechanism of Rhein in the treatment of liver cancer. In this paper, we have provided the first evidence that Rhein promotes apoptosis through regulating ROS/JNK/Jun/caspase-3 signaling pathway.

## Materials and methods

### Drugs

Rhein was purchased from the Chinese National Institute. It was dissolved in DMSO, and was added into the culture medium at the indicated concentrations (with a final DMSO concentration less than 0.1%).

### Cell culture

HepG2 and Huh7 cells were obtained from the Cell Bank of Chinese Academy of Sciences (Shanghai, China). Cells were cultured with DMEM medium containing 10% FBS and antibiotics (100 U/mL penicillin and 100 mg/mL streptomycin) in CO_2_ incubator (at 37°C).

### MTT analysis

Cells were treated with Rhein (0, 50, 100, 150, and 200 μmol/L) and cultured for 24 h, 48h, and 72h, respectively. After exposure to different concentrations of Rhein, the cell viability was detected with MTT analysis. Details of MTT analysis were in compliance with the previously described 7.

### Hoechst staining analysis

Cells were treated with Rhein (0, 100, 150, and 200 μmol/L) for 24 h in 96-well culture plates. Hoechst staining analysis was performed as described previously 8.The stained cells were observed with fluorescence-inverted microscopy (IX73; Olympus, Tokyo, Japan).

### TUNEL staining

Cells were treated with Rhein (0, 100, 150, and 200 μmol/L) for 24 h. For *in situ* apoptosis detection, the cells were stained using TUNEL reagent according to the manufacturer's instructions. TUNEL-positive cells were analyzed under a fluorescence microscope. The data analysis of TUNEL staining was conducted as described previously 9.

### ROS level analysis

ROS level was evaluated using ROS assay kit based on 2',7'-Dichlorodihydrofluorescin diacetate (DCFH-DA). Cells were treated with Rhein (0, 100, 150, and 200 μmol/L) for 24 h, and then incubated with DCFH-DA (50 μmol/L) for 30 min in the dark. ROS level analysis was performed as described previously 8.

### MMP level analysis

MMP level was measured with JC-1 staining. Cells were treated with Rhein (0, 100, 150, and 200 μmol/L) and CCCP (10 μmol/L, as the positive control) for 24 h, respectively. Then, the cells were stained with JC-1 reagent (10 μg/mL) at 37°C for 20 min. The result was analyzed by a flow cytometer (Becton Dickinson, USA). MMP level analysis was performed as described previously 10.

### Apoptosis and cell-cycle arrest analysis

The apoptosis and cell-cycle arrest analysis were performed by FACS. Cells were treated with Rhein (150 μmol/L) or NAC (1 mmol/L) for 24 h, and then were stained by annexin V-APC in conjunction with propidium iodide (PI). The detail of apoptosis and cell-cycle arrest analysis was conducted as described previously 11.

### Western blot analysis

Western blot analysis was conducted as described previously 12. Briefly, the total proteins were extracted with RIPA buffer (Beyotime, China). Protein concentrations were measured using enhanced BCA protein Assay kit (Beyotime, China) by spectrophotometer. Equal amounts of protein (50μg) were separated using 10% sodium dodecyl sulfate polyacrylamide gel electrophoresis (SDS-PAGE), were transferred onto PVDF membrane, and then were blocked with 5% fat-free dry milk at room temperature for 1h. The membranes were incubated with primary antibodies including p-JNK(1:1000), JNK(1:1000), p-c-Jun(1:1000), c-Jun(1:1000), cleaved caspase-3(1:1000), caspase-3(1:1000) and β-actin(1:2000) at 4°C overnight , respectively. The next day, the membranes were washed using TBST washing buffer, and then incubated with the peroxidase-conjugated secondary antibody (1:5000) for 1 h at room temperature. After washed with TBST, the membranes were developed using ECL plus chemiluminescence kit on a DNR bio-imaging system MicroChemi. β-actin was used as an internal control to normalize results. The images were quantified using Image J software.

### Statistical analysis

All data were presented as mean ± SD. The GraphPad prism5.0 Software was used for statistical analysis. Student's *t*-test was used for the comparison between two samples, and two-way ANOVA analysis for two groups. A *P* < 0.05 was considered as statistical significance.

## Results

### Rhein increased the apoptosis of HepG2 and Huh7 cells

To investigate the effects of Rhein on the viability of HepG2 and Huh7 cells, we first examined the cell viability using MTT assay. The cells were treated with Rhein in a concentration gradient (0, 50, 100, 150 and 200 μmol/L) for 24, 48 and 72 h, respectively. As shown in Figure 1B, Rhein significantly increased cell death in a dose-dependent and time-dependent manner. The higher concentration of Rhein for 72 h has a stronger pro-apoptotic effect. These results suggest that Rhein induces cell apoptosis both in HepG2 and Huh7 cells.

To further investigate the effect of Rhein on HepG2 and Huh7 cells, we tested the role of Rhein (0, 100, 150 and 200 μmol/L for 24 h) on apoptotic morphological characteristics with Hoechst 33342 staining. Our data showed that Rhein increased the condensation and fragmentation of nuclei, indicating that the drug enhances cell apoptosis (Figure 1C and D). Altogether, these results suggest that Rhein promotes cell apoptosis of liver cancer cells *in vitro*.

### The effects of Rhein on the MMP

**Figure 2:**
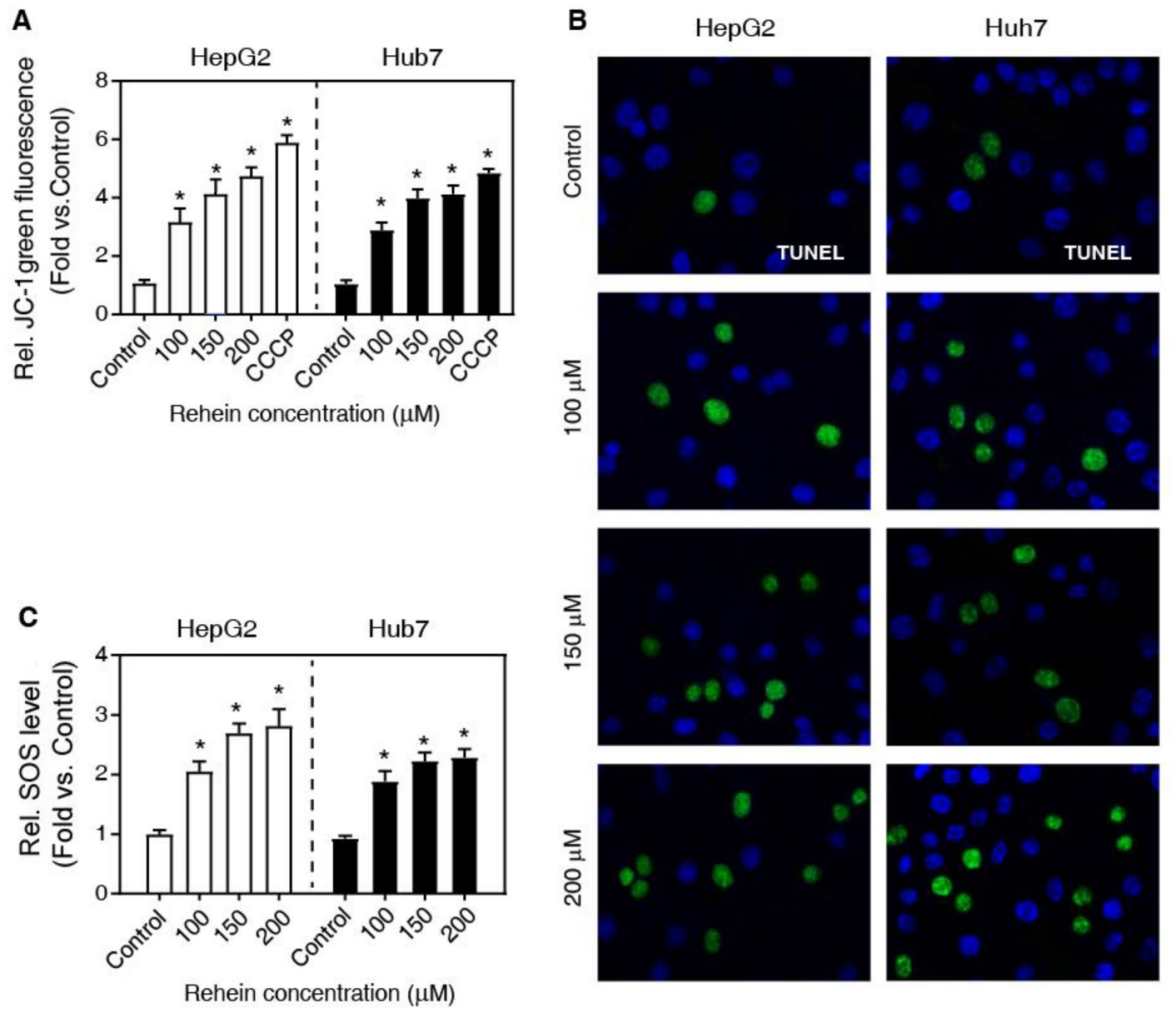
** The effects of Rhein on the MMP. (A)** HepG2 and Huh7 cells were treated with Rhein (0, 100, 150 and 200 μmol/L) or CCCP (10 μmol/L). The MMP was measured with flow cytometer using JC-1 staining. **(B)** HepG2 and Huh7 cells were treated with Rhein (0, 100, 150 and 200 μmol/L) for 24 h, and then the DNA fragmentation was detected using a TUNEL kit. **(C)** HepG2 and Huh7 cells were treated with Rhein (0, 100, 150 and 200 μmol/L) for 24 h. The ROS level was measured with DCFH-DA. *P < 0.05 compared with the control group.

Previously, it has been demonstrated that MMP is one of the main characteristics of early apoptosis of cells 13. To investigate whether mitochondria are involved in Rhein-induced apoptosis, we measured MMP of cells using JC-1 staining. As shown in Figure 2A, Rhein exhibited a significant loss of MMP in a dose-dependent manner compared with the control group, close to the effects of CCCP (a widely used reagent to induce cell apoptosis) treatment. In addition, we also analyzed DNA fragmentation using the TUNEL staining, a widely used method to determine cell apoptosis. Our results showed that the cells of Rhein group exhibited elevated double-stranded DNA breaks compared with the control group both in HepG2 and Huh7 cells (Figure 2B). Altogether, these results suggest that Rhein increases the apoptosis of HepG2 and Huh7 cells.

### Rhein increases the level of ROS

**Figure 3:**
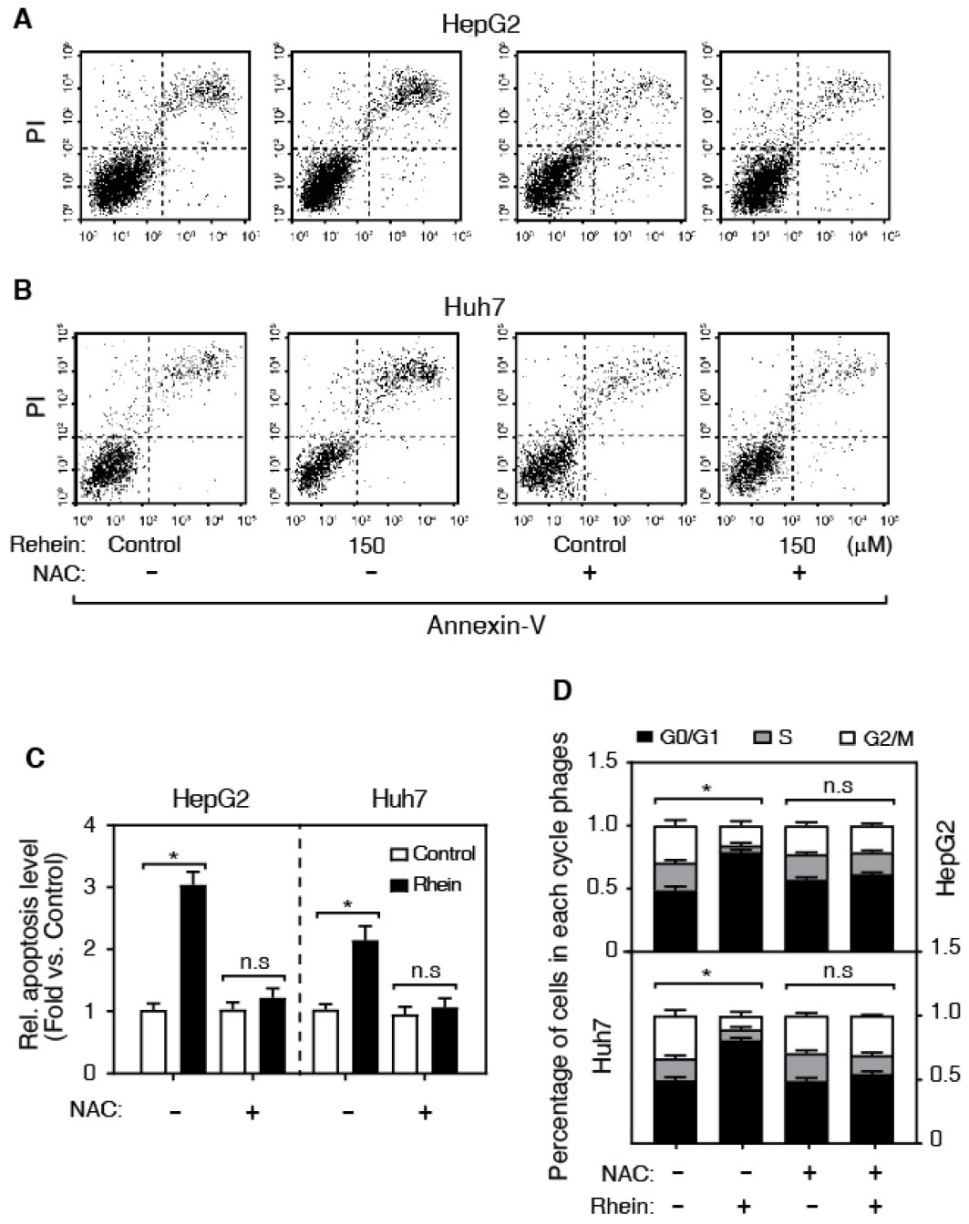
Rhein increases cell apoptosis and cell-cycle arrest. **(A-C)** The effect of NAC on Rhein-induced cell apoptosis. HepG2 and Huh7 cells were treated with control or Rhein, and then incubated with NAC. **(D)** The effect of NAC on Rhein-induced the cell-cycle arrest. *P < 0.05; n.s, not statistically significant.

It is well known that ROS is a pro-apoptosis factor 13, 14. To determine whether ROS is associated with the role of Rhein on apoptosis in HepG2 and Huh7 cells, we examined the regulatory role of Rhein (0, 100, 150 and 200 μmol/L for 24 h) on ROS level using DCFH-DA-based assay. As shown in Figure 2C, Rhein significantly increased the ROS level in a dose-dependent manner, suggesting that Rhein induces the generation of ROS. To confirm that Rhein increases apoptosis through regulating the ROS level, we pre-treated cells with NAC, a widely used ROS scavenger. Interestingly, NAC obviously abolished the cell apoptosis induced by Rhein (Figure 3A-C). Conclusively, these data suggest that Rhein induces apoptosis via regulating the level of ROS in HepG2 and Huh7 cells. Next, we analyzed the effect of Rhein on the cell-cycle progression by FACS. As shown in Figure 3D, Rhein induced a marked G0/G1 arrest both in HepG2 and Huh7 cells, and these effects were abolished by NAC pre-treatment. These results collectively indicate that Rhein induces cell apoptosis and cell-cycle arrest via regulating the ROS level *in vitro.*

### Rhein induced apoptosis via regulating the JNK/Jun/caspase-3 signaling pathway

**Figure 4:**
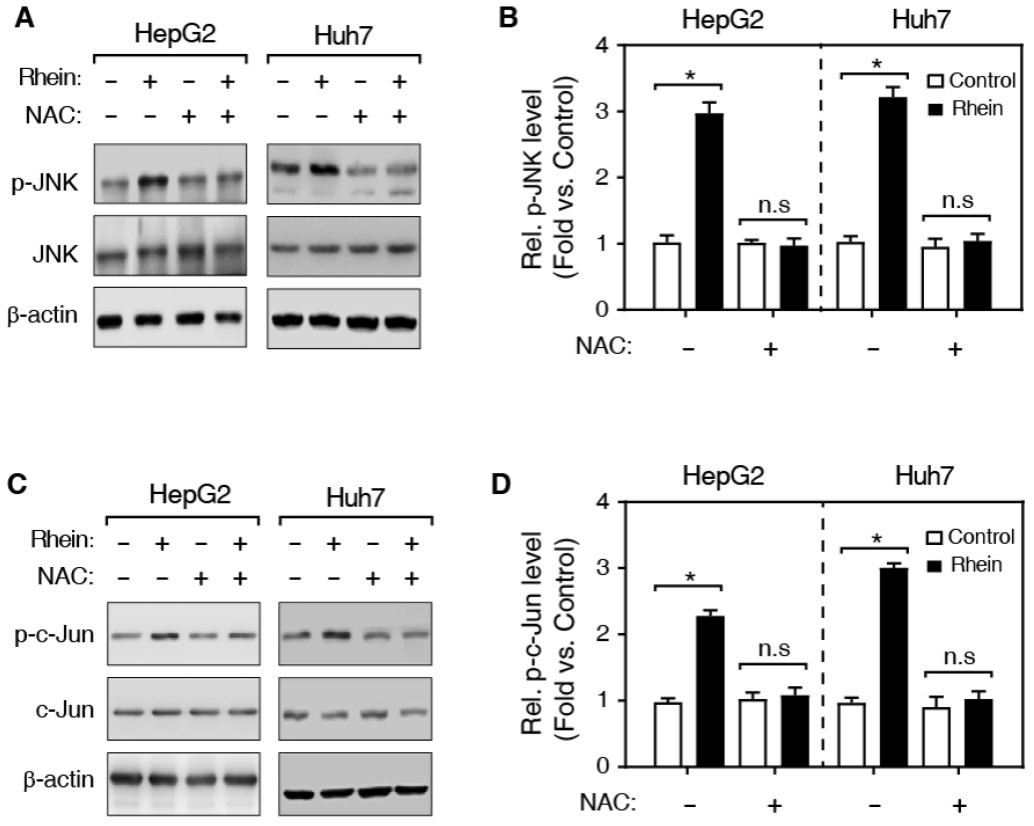
**Effects of Rhein on the phosphorylation of JNK and c-Jun. (A, C)** Western blot determined expression levels of JNK, p-JNK (A) and c-Jun, p-c-Jun (C). HepG2 and Huh7 cells were treated with/without Rhein, and then incubated with/without NAC. β-actin served as the loading control. **(B, D)** The quantification of protein levels in A and C. *P < 0.05; n.s, not statistically significant.

**Figure 5:**
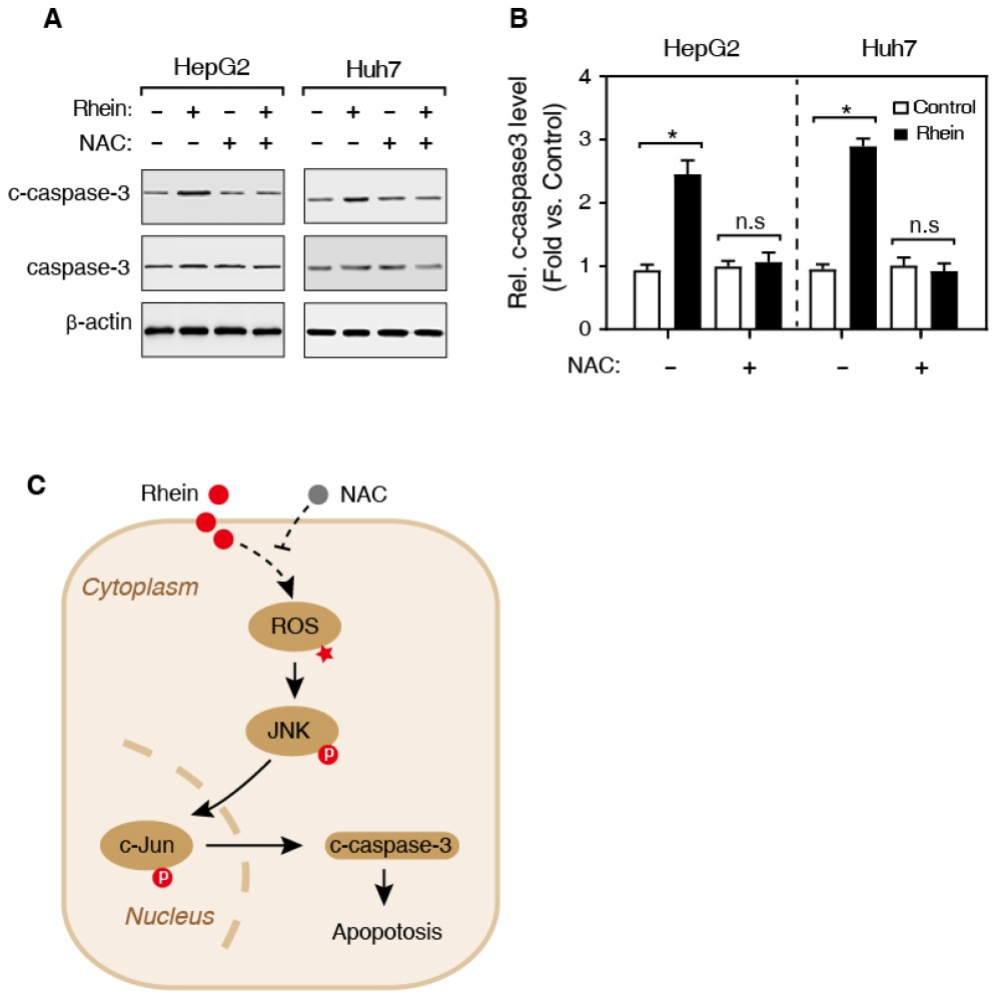
** Effects of Rhein on the expression of cleaved caspase-3. (A)** Western blot determined the expression of caspase-3 and c-caspase-3. HepG2 and Huh7 cells were treated with/without Rhein, and then incubated with/without NAC. β-actin served as the loading control. **(B)** The quantification of the c-caspase-3 protein level in A. *P < 0.05; n.s, no significance in statistic. **(C)** The schematic representative of work model. Rhein induces the generation of ROS, and then activates the JNK/Jun/caspase-3 signaling pathway to promotes cell apoptosis. NAC, N-acetylcysteine; ROS, reactive oxygen species; JNK, c-Jun N-terminal kinase; p, phosphorylation modification.

Previous studies have shown that ROS activates the JNK kinase, and the activated JNK subsequently phosphorylates its substrate, c-Jun, and the phosphorylated c-Jun (p-c-Jun) further induces the activation of caspase-3 protein 15 (Figure 5C). Therefore, we speculated that Rhein induces cell apoptosis by regulating the JNK/Jun/caspase-3 signaling pathway. Then, we firstly detected the expression of phosphorylated JNK (p-JNK) using western blotting. As shown in Figure 4A and B, Rhein increased p-JNK level both in HepG2 and Huh7 cells. Meanwhile, NAC pretreatment significantly blocked the elevated p-JNK level induced by Rhein. Moreover, our findings further showed that Rhein also significantly increased the phosphorylation of c-Jun (p-c-Jun) (Figure 4C and D) and the cleaved caspase-3 protein (c-caspase-3) level (Figure 5A and B), and these effects of Rhein were obviously reversed by NAC pre-treatment. Taken together, our observations suggest that Rhein-induced ROS activates the JNK/Jun/caspase-3 signaling pathway, and then induces cell apoptosis *in vitro*.

## Discussion

Liver cancer is an untreatable solid tumor. After long-term therapy, many patients of advanced liver cancer eventually become treatment-resistant. It was previously reported that the inhibition of cell apoptosis plays a vital role in the pathogenesis of cancers 16. Therefore, promoting cell apoptosis has been a critical strategy for antitumor therapy during the past several decades 17, 18. Emerging evidence indicates that the up-regulation of ROS level is required for the initiation of apoptotic responses induced by several anticancer agents. ROS mediates many physiological and pathological progress in cells via JNK signaling pathway. The activated JNK kinase phosphorylates the c-Jun protein, by which c-Jun initiates the downstream apoptotic events including the cleavage of caspase-3 19-21. Thus, ROS/JNK/c-Jun/caspase-3 signaling has been recognized as an important pathway for developing anticancer drugs.

In the present study, we found that Rhein induced an obvious nuclei condensation and fragmentation (a typical characteristic of apoptotic cells) in HepG2 and Huh7 cells in a dose- and time-dependent manner, indicating Rhein possesses a significant pro-apoptotic effect. Furthermore, our data showed that Rhein induced double-stranded DNA breaks in HepG2 and Huh7 cells, determined by the TUNEL staining. We also observed that Rhein induced early cell apoptosis, indicated by a significant loss of MMP *in vitro*. These findings suggest that Rhein increases cell apoptosis both in HepG2 and Huh7 cells. Meanwhile, we determined the ROS level in cells, and found that Rhein significantly increased ROS level in HepG2 and Huh7 cells. More importantly, scavenging ROS by NAC pre-treatment obviously alleviated the cell apoptosis induced by Rhein. Besides, Rhein induced a marked G0/G1 arrest in HepG2 and Huh7 cells, which also was reversed by NAC pre-treatment. In brief, these results demonstrate that Rhein induces cell apoptosis and cell-cycle arrest by regulating ROS level. In our knowledge, we demonstrated, for the first time, that Rhein induces cell apoptosis in the context of liver cancer cells.

It was previously reported that ROS level in cells affects the activity of many signaling pathways, including JNK signaling pathway 22. The activated JNK kinase phosphorylates c-Jun protein, and finally leads to the cell apoptosis in a caspase-3 dependent manner 23. In this study, we firstly found that Rhein increased the phosphorylation of JNK both in HepG2 and Huh7 cells. At the same time, NAC significantly inhibited the over-expression of p-JNK induced by Rhein. Also, our result showed that Rhein significantly increased the p-c-Jun and the c-caspase-3 protein, and the effects of Rhein were obviously reversed by NAC pre-treatment both in HepG2 and Huh7 cells. These results suggest that Rhein-induced ROS activates the JNK/Jun/caspase-3 signaling pathway, and then induces apoptosis *in vitro*.

In fact, some previous studies reported that Rhein increases the expression of p53, p21/WAF1, CD95, and its two forms of ligands in HepG2 cells. Besides, Rhein also reduces the expression of Bcl-2 and Bcl-XL, and increases the expression of Bax and Bak. Many independent groups have also reported that Rhein activates caspase-1, -3, -8, -9, and -12. Altogether, these results suggest that the roles of Rhein in antitumor involve multiple pathways, and the pharmacological mechanisms of Rhein in liver cancer need further studies.

Taken together, our observations demonstrated that Rhein increases apoptosis through inducing the generation of ROS, and then activates JNK/Jun/caspase-3 signaling (Figure 5C). Overall, the present study has provided the first evidence that Rhein promotes apoptosis via regulating ROS/JNK/Jun/caspase-3 signaling pathway both in HepG2 and Huh7 cells. These results may contribute to understanding the roles of Rhein in apoptosis, and developing new therapeutic approaches for liver cancer treatment.

## Abbreviations

ROS: reactive oxygen species
NAC: N-acetylcysteine
MMP: mitochondrial membrane potential
JNK: c-Jun N-terminal kinase
